# Investigating Tissue Optical Properties and Texture Descriptors of the Retina in Patients with Multiple Sclerosis

**DOI:** 10.1371/journal.pone.0143711

**Published:** 2015-11-30

**Authors:** Boglárka Enikő Varga, Wei Gao, Kornélia Lenke Laurik, Erika Tátrai, Magdolna Simó, Gábor Márk Somfai, Delia Cabrera DeBuc

**Affiliations:** 1 Department of Ophthalmology, Faculty of Medicine, Semmelweis University, Budapest, Hungary; 2 Bascom Palmer Eye Institute, University of Miami, Miller School of Medicine, Miami, Florida, United States of America; 3 Department of Neurology, Faculty of Medicine, Semmelweis University, Budapest, Hungary; Justus-Liebig-University Giessen, GERMANY

## Abstract

**Purpose:**

To assess the differences in texture descriptors and optical properties of retinal tissue layers in patients with multiple sclerosis (MS) and to evaluate their usefulness in the detection of neurodegenerative changes using optical coherence tomography (OCT) image segmentation.

**Patients and Methods:**

38 patients with MS were examined using Stratus OCT. The raw macular OCT data were exported and processed using OCTRIMA software. The enrolled eyes were divided into two groups, based on the presence of optic neuritis (ON) in the history (MSON+ group, n = 36 and MSON- group, n = 31). Data of 29 eyes of 24 healthy subjects (H) were used as controls. A total of seven intraretinal layers were segmented and thickness as well as optical parameters such as contrast, fractal dimension, layer index and total reflectance were measured. Mixed-model ANOVA analysis was used for statistical comparisons.

**Results:**

Significant thinning of the retinal nerve fiber layer (RNFL), ganglion cell/inner plexiform layer complex (GCL+IPL) and ganglion cell complex (GCC, RNFL+GCL+IPL) was observed between study groups in all comparisons. Significant difference was found in contrast in the RNFL, GCL+IPL, GCC, inner nuclear layer (INL) and outer plexiform layer when comparing MSON+ to the other groups. Higher fractal dimension values were observed in GCL+IPL and INL layers when comparing H vs. MSON+ groups. A significant difference was found in layer index in the RNFL, GCL+IPL and GCC layers in all comparisons. A significant difference was observed in total reflectance in the RNFL, GCL+IPL and GCC layers between the three examination groups.

**Conclusion:**

Texture and optical properties of the retinal tissue undergo pronounced changes in MS even without optic neuritis. Our results may help to further improve the diagnostic efficacy of OCT in MS and neurodegeneration.

## Introduction

Multiple sclerosis (MS) is a neurodegenerative disorder, a chronic inflammatory process that affects the central nervous system (CNS) by the demyelination of axons of the brain and spinal cord. It is the most common disease of the CNS that causes permanent disability in young adults. [1] The non-myelinated parts of the axons of the retinal ganglion cells, the retinal nerve fiber layer (RNFL) provides an optimal opportunity to examine the neuronal loss caused by demyelination.

Optical coherence tomography (OCT) is a non-invasive, non-contact, high-resolution imaging modality that is capable of capturing cross-sectional images of the retina. [2] It is based on the optical reflectance differences of the analyzed tissue and thus it may be applied in the detection of pathological retinal changes. [3] OCT technology has gained very wide use in ophthalmology and it is stipulated that it is one of the most frequently used decision making techniques in the field. [4] The use of custom-built algorithms for the segmentation of retinal OCT images may enable the detection and follow-up of early neural loss in patients with MS. [5–8] It has also been postulated that the inner nuclear layer (INL) is possibly also involved in the pathological processes in MS, with different observations describing microcystic edema of the INL and also the thickness changes of the INL. [9, 10]

Although thickness differences may discern regions with signs of retinal disease from normal regions, differences in texture descriptors of normal and abnormal retinal tissue may also provide additional information of disease development. In fact, the appropriateness of texture to classify tissues in OCT images has been shown in previous studies. [11] By analyzing the spatial arrangement of color or intensities in an image or selected region of interest (ROI), the image irregularities can be measured. Consequently, texture features, such as contrast and fractal dimension could be analyzed for the macula and each intraretinal layer. The fractal dimension (FD) of a profile or surface is a roughness measure regarded as a local property of the system with higher values indicating rougher surface. [12] There are different methods to determine the FD. The typical conventional approach used to calculate the fractal dimension of an image is the box-counting method but the power spectrum method is demonstrated to be more robust. [13, 14]

Finally, as mentioned above, the most common parameter investigated during the OCT examination is retinal thickness. In reality, reflectance is the direct measurement from which thickness is calculated in OCT systems. The human retina is an almost transparent tissue that only reflects about 1% of the incident light. [15] Retinal tissue is characterized by many small random fluctuations in refractive index caused by the ultrastructure of the tissue. [16] As a consequence, incident light on tissue is deflected or scattered off this structure. Therefore, differences in optical properties of normal and abnormal retinal tissue may also provide additional information of disease development in pathological eyes allowing OCT technology to be used for quantitative analysis of tissue optical properties. [17–19] Accordingly, Bizheva et al have shown previously that optical properties of the retina may change due to their metabolic activity. They were using optical coherence tomography for this purpose and named the method optophysiology. [20] Huang et al. have shown the early changes of reflectance of the RNFL in a rat model of glaucoma preceding the pathological changes in the retina. [21] We have shown previously that diabetes not only causes thinning of the inner retinal layers, but also reduces the amplitude of the back-reflected signal from these layers. [22, 23] Consequently, diagnostic predictors based on reflectance changes may be of interest in multiple sclerosis as well where pathological processes of the inner retina have been well described previously.

In this study, the differences in texture and optical properties of the retinal tissue in patients with MS compared to healthy subjects are evaluated using OCT image segmentation in order to investigate their usefulness in the detection of retinal neurodegenerative changes. Significant differences in the nerve fiber layer and the ganglion cell/plexiform layer complex are observed in MS patients compared to healthy subjects and in eyes affected by optic neuritis compared to those unaffected. Our findings may help to further improve the diagnostic efficacy of OCT in MS and neurodegeneration.

## Patients and Methods

### Study Population and examinations

All participants were treated in accordance with the tenets of the Declaration of Helsinki. Institutional Review Board approval was obtained for all study protocols (Semmelweis University Regional and Institutional Committee of Sciences and Research Ethics). Written informed consent was obtained from all participants in this study. Thirty-eight patients with relapsing-remitting multiple sclerosis were enrolled from the Department of Neurology of Semmelweis University. The clinical data, including disease duration, medical treatment and the date of MSON episodes was compiled as part of a complete neurological examination performed by a board-certified neurologist. Based on the neurological, functional, elecrophysiological, radiological and laboratory examinations, all ON episodes were diagnosed as MS associated ON and all patients were meeting the revised McDonald criteria. [24] The control group (H) was made of twenty-nine randomly selected eyes of twenty-four age-matched healthy controls.

Each subject underwent routine ophthalmic examination including best corrected visual acuity measurement, applanation tonometry, critical flicker frequency assessment (CFF) and slit-lamp examination. All study subjects were assessed by the same, expert and trained operator with a Stratus OCT device (Carl Zeiss Meditec Inc., Dublin, CA, USA) using the “macular map protocol”, which consists of six fovea centered scan lines in radial directions, each having a 6 mm transverse length. One study eye per each patient was selected randomly.

In order to ensure quality control of the OCT scans included and segmented in the study, we used the scan quality factor (SQF) criteria in OCTRIMA for checking the scan variance using the foveal center point thickness as reported previosuly [25] and also adapted the OSCAR-IB system described by Tewarie at al. [26]

The scan quality factor (SQF) is based on the standard deviation calculation (in percent) of the center point thickness (CPT) for the six radial line scans included in the OCTRIMA software and is used to control the variability of measurements associated to image acquisition pitfalls. A high standard deviation (>10% of center point thickness) means high variability, usually due to patient movement or boundary line error, thus leading to incorrect center point thickness values. [25, 27] We only included scans with a SQF = 1, indicating that the percentage standard deviation of the foveal center point was ≤10%.

Furthermore, using the OSCAR-IB system the following details were observed and evaluated on each set of scans to be included in the study: Obvious problems not covered by items below; Sufficient OCT Signal (SS>6, [25]); Foveal centered scans (as a second pass of quality control); Algorithm failure; Retinal pathology impairing the segmentation; Proper fundus illumination; and Measurement beam placed centrally (no tilting of the B-scans). The exclusion criteria were similar to those applied by Tewarie et al., including the following: (1) spherical or cylindrical correction higher than 3.0 diopters, (2) the presence of any media opacities (corneal pathologies, cataract, floaters, etc.) (3) the presence of any retinal disease or optic neuropathy including glaucoma, except ON, (4) intraocular pressure higher than 20 mmHg in the medical history, (5) previous eye surgery, (6) amblyopia, (7) last MSON episode less than 6 months prior to enrollment, (8) bad fixation cooperation during the OCT examination (e.g. due to nystagmus) and (9) low signal strength of the OCT images (SS≤6). None of the patients were under fingolimod treatment before and during the examination period. [28] As in previous TD-OCT studies [29–31] where highly repeatable measurements have been obtained with signal intensity scores of at least 6 to 7, we excluded images with signal strength SS≤6 as we have previously shown this is the threshold for repeatable segmentation analysis with OCTRIMA. [25]

The eligibility criteria for control subjects were best-corrected Snellen visual acuity of 20/20 and the lack of any ocular or systemic diseases, with good quality OCT scans as described above.

### OCT image processing

**Fig 1:**
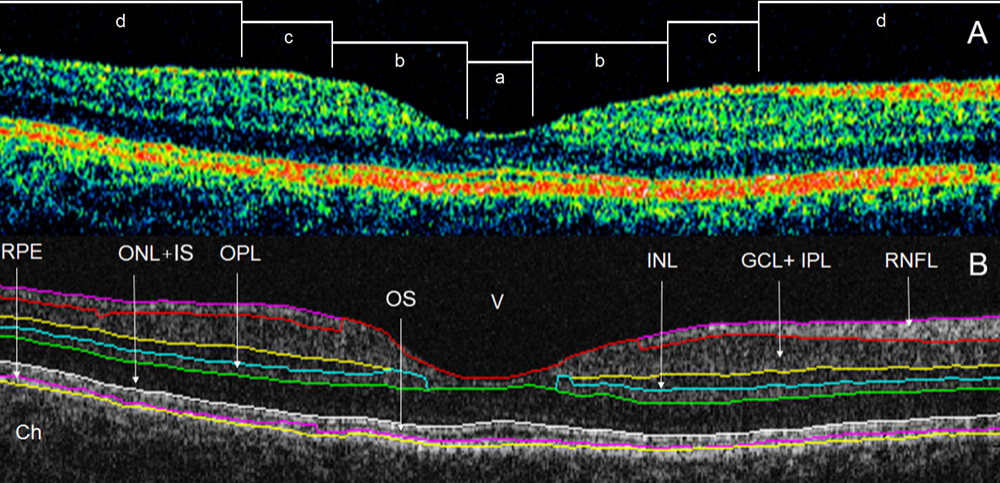
Macular image segmentation results using OCTRIMA. (A) The image of a healthy macula scanned by Stratus OCT with the division of the macular regions used for the analysis (foveolar region (a) with a diameter of 0.375 mm, foveal region (b) with a diameter of 1.85 mm; parafoveal region (c) with a diameter of 2.85 mm and perifoveal region (d) with a diameter of 5.85 mm) (B) The same OCT scan processed with OCTRIMA. Abbreviations: Ch, choroid; GCL+IPL, ganglion cell layer and inner plexiform layer complex; INL, inner nuclear layer; ONL+IS, combined outer nuclear layer and inner segment of photoreceptors; OS, outer segment of photoreceptors; OPL, outer plexiform layer; RNFL, retinal nerve fiber layer; RPE, retinal pigment epithelial layer; V, vitreous.

The radial scans were exported and processed by a custom-built software (OCTRIMA) developed by Cabrera et al. [6]. This OCT image segmentation program is capable of segmenting 7 cellular layers of the retina based on their optical densities (see Fig 1): the RNFL, the ganglion cell and inner plexiform layer complex (GCL+IPL), the INL, the outer plexiform layer (OPL), the outer nuclear layer and inner photoreceptor segment (ONL+IS), outer photoreceptor segment (OS) and retinal pigment epithelium (RPE). [6] Because of the special arrangement of the retinal structure below the foveal pit where the inner retinal layers are displaced concentrally, the segmentation of the foveolar region was limited to the three outer retinal layers (ONL+IS, OS, RPE).

The OCTRIMA software integrates a denoising and edge enhancement technique (also removing the speckle noise) along with a segmentation algorithm. [6] In our previous works the high repeatability and reproducibility of OCTRIMA measurements in healthy subjects was found. The reproducibility was the highest for the thickness measurements of the ONL, ganglion cell complex (GCC, composed by the RNFL and GCL+IPL), GCL+IPL and RNFL, the inter- and intraexaminer, intervisit variabilities being around the resolution of currently available OCT devices for all layers (<6 μm for all layers and all comparisons). [25] It is of note that total retinal thickness is measured between the inner limiting membrane and the inner boundary of the photoreceptor outer segment/RPE junction by this software. It is also important to mention that ONL is a thin membrane, which contains both the external limiting membrane and the inner segment of the photoreceptors. This layer cannot be visualized clearly in Stratus OCT images which makes the segmentation of the inner segment more difficult. Thus, the segmentation of this layer does not reflect the actual anatomic structure.

The eyes of the MS patients were divided into two study groups for further analyses. The first group was composed of 36 eyes, which had MS-associated ON at least 6 months prior to enrollment (MSON+). 31 eyes without ON episode (MSON-) in the history formed the second group. The diagnosis of optic neuritis was based on the patient's medical history. An acute episode of MSON was defined by clinical symptoms such as decreased visual acuity developing in few days, pain on eye movement, abnormal response on visual evoked potential examination confirming prechiasmal lesion and decrease in the CFF. The CFF is a basic examination of optic nerve function, and plays an important role in the diagnosis and also in the follow-up of ON. Decreased CFF is a common characteristic of an acute ON episode, and it also can be affected after the recovery both of which can help to establish the diagnosis of ON. In most cases a good recovery was observed within 2–3 months. In the subjects with ON appearing as a first symptom, routine MRI examinations showed characteristic signs of MS and confirmed the diagnosis. Demographic and clinical characteristics including age, gender, duration of disease, elapsed time between the last MSON episode and OCT examination and best corrected visual acuity are shown in Table 1.

**Table 1:** Descriptive statistics of the study participants.

	Healthy group	MSON- group	MSON+ group
Number of eyes	29	31	36
Number of subjects *(female/male)*	24 (16/8)	25 (14/11)	26 (19/7)
Age*(years*, *mean ±SD*, *[median])*	33 ± 9 [31]	34 ± 9 [35]	34 ± 9 [35]
BCVA *(mean ±SD*, *[median])*	1.0 ± 0.00 [1.0]	1.0 ± 0.00 [1.0]	0.91 ± 0.25 [1.0]
Disease duration*(months*, *mean ±SD [median])*	N/A	71 ± 51 [48]	72 ± 51 [53]
Elapsed time between last MSON episode and examination date *(months*, *mean ± SD [median])*	N/A	N/A	49 ± 38 [38]

Abbreviations: SD (standard deviation); H (Healthy subjects group); MSON- (eyes of patients with multiple sclerosis without optic neuritis in medical history); MSON+ (eyes of patients with multiple sclerosis with optic neuritis in medical history); BCVA (best corrected visual acuity).

### Retinal layer thickness, texture and optical measurements on OCT images

In order to evaluate the diagnostic power of optical properties and texture descriptors compared to standard thickness measurements, mean thickness values per intraretinal layer were extracted by computation of the mean distance between the borders of each layer. We note that we removed the noise first and then performed the processing so that noise effect could be nullified. Therefore, all calculations were performed after removing the speckle noise from raw OCT data using a complex diffusion filter. [6] We also note that factors extrinsic to the retina, such as media opacity, poor focusing, and scanning pitfalls, were removed by taking the ratio of the average reflectivity signal within the retina and each intraretinal layers and dividing it by the average reflectivity signal from a reference layer. Accordingly, the analyses performed using parameters based on optical properties and tissue descriptors considered mean reflectivity values that were calculated using reflectivity with normalization to the RPE reflectance (NRPE). [32] The mean values were calculated to each layer across the six radial OCT scans. [33]

A method based on the power spectrum was used to calculate the fractal dimension in OCT images. [14] Since the average power spectrum of an image obeys a power law scaling, the fractal dimension was calculated from the power law detected in the graph of the power spectrum as a function of the frequency in the Fourier transform of the OCT image (gray scale). In this particular case, when the graph is plotted in a log-log scale the curve is approximately similar to a straight line and the dimension is provided by the slope of the line.

The fast Fourier transform (FFT) was applied to the OCT reflectance’s profiles to obtain the power spectrum as follows:

$$P(\omega )∼\omega^{-\beta }$$ (1)

where P(ω) is the power spectrum with the frequency ω. β is the spectral exponent of the reflectance profile. The Eq (1) can be converted into:

ln(P(ω))∼−βln(ω) (2)

The fractal dimension is linked to the power-law exponent by the following relationship: [14]

$$FD=\frac{5-\beta }{2}$$ (3)

Therefore, the fractal dimension was evaluated from the slope β of a least-square regression line fit to the data points in log-log plot of power spectrum. The fractal dimension was calculated for the reflectance profile within each intraretinal layer for each A-scan. The mean value of the fractal dimension was calculated by averaging the fractal dimension measurements across all A-scans in each macular region of each intraretinal layer. MATLAB software (The Mathworks, Natick, MA) was used to perform the fractal dimension analysis using a custom-built algorithm. Contrast measures were extracted by using second-order statistical texture analysis.

Optical properties such as total reflectance and layer index has been described previously and calculated as follows. [23, 33, 34] Total reflectance *TR*_*k*_ was calculated by summarizing reflectivity values of the elements in the column *k* of the selected ROI.

$$TR_{k}=MR_{k}\times \frac{H_{k}}{\Delta y}$$ (4)

where *MR*_*k*_ is the mean reflectance in the column *k* of the selected ROI, *H*_*k*_ is the local thickness of ROI in the column of *k*, and Δ*y* is the pixel resolution defined by the OCT device.

Layer index *LI*_*k*_ was defined as:

$$LI_{k}=MR_{k}\times \frac{H_{k}}{I_{sa}}=\frac{1}{N_{k}}\sum_{j=1}^{N_{k}}I_{j,k}\times \frac{H_{k}}{I_{sa}}$$ (5)

where *MR*_*k*_ is the mean reflectance in the column *k* of the selected ROI, *H*_*k*_ is the local thickness of ROI in the column of *k*. *I*_*sa*_ is the reflectivity value representing the *99%* of all recorded reflectivity values in a given retinal OCT image.

**Fig 2:**
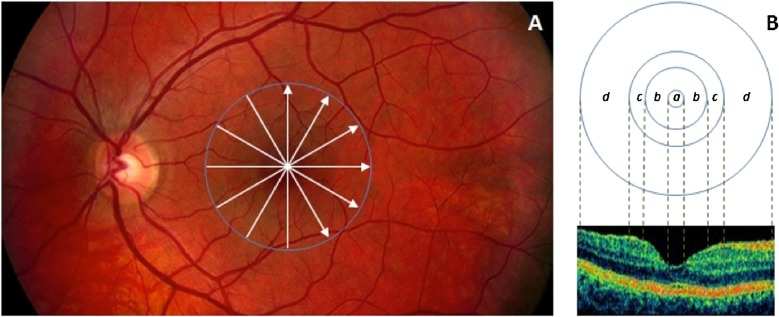
Retinal scanning and macular regions used in the study. (A) The fundus image of a healthy eye. The white arrows in the macula show the locations of the OCT scans made. (B) The distribution of macular regions: foveolar region (a) with the diameter of 0.375 mm, foveal region (b) with a diameter of 1.85 mm; parafoveal region (c) with a diameter of 2.85 mm and perifoveal region (d) with a diameter of 5.85 mm.

It is worth to note that before the calculation of parameters related to reflectance values, the lateral coordinates of the blood vessel shadows were obtained by the help of a blood vessel shadowgram technique and were removed in each OCT image. [35] Following this, these shadows were removed in each OCT image before calculating the values for reflectance. Total reflectance values included average values of reflectance with normalization to the RPE reflectance (NRPE). Average values of total reflectance per intraretinal layer were calculated. Total reflectance values were converted to decibels (dB = 10 ×log10 [TR]). Layer index parameter was based on the mean reflectance corrected to the thickness of the region of interest and the 99% of the total reflectance of the scan (see details above and Eq 5). [18] All calculated values were expressed in the foveolar, foveal, parafoveal and perifoveal regions and also as a mean across all macular regions. (Fig 2.) In the foveolar region, the calculations were limited to the outer retinal layers (OPL+IS, OS, RPE). For the analyses an additional composite layer was defined, the GCC consisting of the RNFL and GCL+IPL layers with the purpose to observe the changes of the whole ganglion cell with its proximal and distal denrites.

### Statistical analyses

In order to correct for inter-eye bias, mixed model ANOVA analysis was used for the comparison of thickness and optical parameters among the groups. Statistical analyses were performed using the software package SPSS version 16 (SPSS Inc, Chicago, Illinois). A modified p value (p<0.001) was considered as statistically significant.

## Results

### Thickness

The thinning of the RNFL, GCL+IPL and also GCC was significant in all comparisons in each macular region except the foveal region. In the foveal region significant difference was observed in GCC between H, MSON- and MSON+ groups, in RNFL between H and MSON+, and GCL+IPL in MSON+ compared to the others. (Table 2.)

**Table 2:** Distribution statistics of the thickness (μm) of intraretinal layers by study group, represented as means ±SD.

Thickness	Healthy	MSON-	MSON+
	H vs. MSON+	H vs. MSON-	MSON+ vs MSON-
**Across All Macular Regions**			
RNFL	38.16 ± 0.55 ‡	35.44 ± 0.54 ‡	32.32 ± 0.54 ‡
GCL+IPL	76.06 ± 1.44 ‡	67.87 ± 1.40 ‡	58.1 ± 1.40 ‡
GCC	114.23 ± 1.85 ‡	103.28 ± 1.80 ‡	90.45 ± 1.95 ‡
INL	35.19 ± 0.35	35.38 ± 0.35	35.26 ± 0.36
OPL	41.70 ± 0.58 *	41.58 ± 0.55	39.92 ± 0.59 *
ONL+IS	86.23 ± 1.21 *	88.20 ± 1.11	89.62 ± 1.19
OS	16.48 ± 0.59	16.58 ± 0.57	16.28 ± 0.62
RPE	13.15 ± 0.18	13.58 ± 0.17	13.21 ± 0.19
**Foveolar Region**			
ONL+IS	123.55 ± 1.79	124.46 ± 1.65	123.57 ± 1.76
OS	26.02 ± 0.62	25.93 ± 0.60	25.21 ± 0.65
RPE	16.10 ± 0.30	16.57 ± 0.28	15.83 ± 0.31 *
**Foveal Region**			
RNFL	12.42 ± 0.45 ‡	10.60 ± 0.42 *	9.7 ± 0.45 *
GCL+IPL	51.59 ± 1.04 ‡	47.51 ± 1.01 *	40.1 ± 1.10 ‡
GCC	63.92 ± 1.28 ‡	58.07 ± 1.24 *	49.9 ± 1.34 ‡
INL	21.00 ± 0.36	20.82 ± 0.34	20.19 ± 0.37
OPL	62.35 ± 1.53 *	61.47 ± 1.44	56.34 ± 1.55 *
ONL+IS	99.45 ± 1.47 *	101.70 ± 1.37	104.43 ± 1.48
OS	18.45 ± 0.79	18.87 ± 0.77	18.73 ± 0.83
RPE	13.41 ± 0.25	13.86 ± 0.25	13.41 ± 0.27
**Parafoveal Region**			
RNFL	34.35 ± 0.53 ‡	31.92 ± 0.52‡	28.96 ± 0.56 ‡
GCL+IPL	93.66 ± 1.86 ‡	82.75 ± 1.81 ‡	69.88 ± 1.97‡
GCC	128.03 ± 2.22‡	114.62 ± 2.16‡	98.92 ± 2.35‡
INL	39.93 ± 0.47	40.59 ± 0.45	40.48 ± 0.49
OPL	37.51 ± 0.53	37.26 ± 0.51	37.23 ± 0.55
ONL+IS	84.54 ± 1.40 *	86.87 ± 1.30	88.44 ± 1.39
OS	14.74 ± 0.72	15.17 ± 0.70	15.04 ± 0.76
RPE	12.64 ± 0.22	12.98 ± 0.21	12.64 ± 0.23
**Perifoveal Region**			
RNFL	43.16 ± 0.69 ‡	39.39 ± 0.68 ‡	35.6 ± 0.73 ‡
GCL+IPL	66.28 ± 1.38 ‡	58.68 ± 1.33 ‡	50.68 ± 1.44 ‡
GCC	16.48 ± 0.59 ‡	98.06 ± 1.79 ‡	86.29 ± 1.95 ‡
INL	38.16 ± 0.55	33.19 ± 0.33	33.04 ± 0.36
OPL	76.06 ± 1.44	31.90 ± 0.27	31.56 ± 0.29
ONL+IS	35.19 ± 0.35	76.50 ± 1.02	77.40 ± 1.10
OS	41.70 ± 0.58	14.57 ± 0.52	14.25 ± 0.56
RPE	86.23 ± 1.21	13.26 ± 0.17	12.93 ± 0.18

Abbreviations: SD (standard deviation); H (Healthy subjects group); MSON- (eyes of patients with multiple sclerosis without optic neuritis in medical history); MSON+ (eyes of patients with multiple sclerosis with optic neuritis in medical history); GCC (ganglion cell complex. the RNFL and GCL+IPL layers together)

* 0.001<p<0.05 and

‡ p<0.001 (Mixed-model analysis ANOVA) between H and MSON+ (see H column). H and MSON- (see MSON- column) and between MSON- and MSON+ (see MSON+ column)

### Contrast

Significantly higher contrast values were observed in the MSON+ group compared to the H and MSON- groups in the RNFL, GCL+IPL, INL, OPL and GCC layers, in the whole macular region. A significant difference was found in the foveal region between MSON+ and the two other groups in the GCL+IPL and GCC, similarly to the INL in the MSON+ versus the healthy group. The GCL+IPL was significantly different between the three groups in the parafoveal region, and so was the RNFL and GCC in the MSON+ vs. H comparison. The perifoveal region showed a significant difference between the three groups in the RNFL, GCL+IPL and GCC layers. (Table 3.)

**Table 3:** Distribution statistics of the contrast (a.u) of intraretinal layers by study group, represented as means ±SD.

Contrast	Healthy	MSON-	MSON+
	H vs. MSON+	H vs. MSON-	MSON+ vs MSON-
**Across All Macular Regions**			
RNFL	1562 ± 26 ‡	1663 ± 25 *	1820 ± 27 ‡
GCL+IPL	1531 ± 21 ‡	1587 ± 21	1727 ± 22 ‡
GCC	3093 ± 43 ‡	3251 ± 42 *	3546 ± 46‡
INL	1394 ± 31 ‡	1428 ± 30	1577 ± 32 ‡
OPL	1314 ± 21 ‡	1325 ± 20	1426 ± 21 ‡
ONL+IS	851 ± 15	862 ± 14	839 ± 15
OS	3506 ± 70	3561 ± 67	3650 ± 73
RPE	5264 ± 82	5222 ± 80	5353 ± 86
**Foveolar Region**			
ONL+IS	881 ± 28	892 ± 27	820 ± 30
OS	3077 ± 67	3118 ± 65	3184 ± 71
RPE	7465 ± 113	7436 ± 111	7742 ± 120 *
**Foveal Region**			
RNFL	2169 ± 88 *	2378 ± 84	2539 ± 91
GCL+IPL	2503 ± 42 ‡	2640 ± 41 *	2935 ± 45 ‡
GCC	4673 ± 106 ‡	5023 ± 102 *	5469 ± 111 ‡
INL	2093 ± 77 ‡	2362 ± 75 *	2477 ± 82
OPL	2052 ± 38	2164 ± 37	2208 ± 40
ONL+IS	960 ± 21	962 ± 20	906 ± 22 *
OS	4120 ± 117	4081 ± 114	4147 ± 124
RPE	7452 ± 114	7426 ± 111	7511 ± 118
**Parafoveal Region**			
RNFL	4122 ± 97 ‡	4366 ± 95	4628 ± 103
GCL+IPL	1872 ± 43 ‡	2144 ± 42 ‡	2387 ± 46 ‡
GCC	5995 ± 120 ‡	6516 ± 118 *	7003 ± 128 *
INL	3879 ± 51	3819 ± 50	3853 ± 54
OPL	4144 ± 52	4131 ± 51	4115 ± 55
ONL+IS	1120 ± 28	1142 ± 27	1093 ± 29
OS	4202 ± 250	4272 ± 243	4245 ± 264
RPE	8150 ± 181	8277 ± 176	8387 ± 187
**Perifoveal Region**			
RNFL	2213 ± 44 ‡	2429 ± 43 ‡	2646 ± 46 ‡
GCL+IPL	1879 ± 39 ‡	2070 ± 38 ‡	2290 ± 41 ‡
GCC	4093 ± 79 ‡	4499 ± 77 ‡	4934 ± 83 ‡
INL	3511 ± 45	3425 ± 43	3477 ± 47
OPL	4038 ± 46	3928 ± 44	4005 ± 47
ONL+IS	1295 ± 27	1282 ± 25	1261 ± 27
OS	4690 ± 154	4830 ± 150	4913 ± 162
RPE	7974 ± 113	7843 ± 111	7848 ± 120

Abbreviations: SD (standard deviation); H (Healthy subjects group); MSON- (eyes of patients with multiple sclerosis without optic neuritis in medical history); MSON+ (eyes of patients with multiple sclerosis with optic neuritis in medical history); GCC (ganglion cell complex. the RNFL and GCL+IPL layers together)

* 0.001<p<0.05 and

‡ p<0.001 (Mixed-model analysis ANOVA) between H and MSON+ (see H column). H and MSON- (see MSON- column) and between MSON- and MSON+ (see MSON+ column)

### Fractal dimension

Fractal dimension was significantly higher in the MSON+ group compared to the H and MSON- groups in the GCL+IPL and INL in the whole macular region, in the INL in the foveal and in the GCL+IPL in the perifoveal regions. Also, a significant difference was found in the OPL between the H and MSON- groups in the parafoveal region. There were no significant differences found in any other layers in other comparisons. (Table 4.)

**Table 4:** Distribution statistics of the fractal dimension (a.u) of intraretinal layers by study group, represented as means ±SD.

Fractal dimension	Healthy	MSON-	MSON+
	H vs. MSON+	H vs. MSON-	MSON+ vs MSON-
**Across All Macular Regions**			
RNFL	1.621 ± 0.009 *	1.637 ± 0.009	1.655 ± 0.009
GCL+IPL	1.699 ± 0.002 ‡	1.695 ± 0.002	1.690 ± 0.002 *
GCC	3.321 ± 0.008 *	3.332 ± 0.008	3.344 ± 0.008
INL	1.779 ± 0.002 ‡	1.785 ± 0.002 *	1.788 ± 0.002
OPL	1.508 ± 0.002 *	1.502 ± 0.002 *	1.503 ± 0.002
ONL+IS	1.778 ± 0.006	1.788 ± 0.006	1.794 ± 0.006
OS	1.701 ± 0.003 *	1.712 ± 0.003 *	1.712 ± 0.004
RPE	1.669 ± 0.002	1.666 ± 0.002	1.668 ± 0.002
**Foveolar Region**			
ONL+IS	1.718 ± 0.008	1.721 ± 0.007	1.731 ± 0.008
OS	1.730 ± 0.004	1.739 ± 0.004	1.736 ± 0.004
RPE	1.642 ± 0.001	1.641 ± 0.001	1.644 ± 0.002
**Foveal Region**			
RNFL	2.005 ± 0.018 *	2.058 ± 0.017 *	2.075 ± 0.018
GCL+IPL	1.931 ± 0.002	1.927 ± 0.002	1.929 ± 0.002
GCC	3.937 ± 0.017 *	3.985 ± 0.016 *	4.003 ± 0.017
INL	2.018 ± 0.004 ‡	2.031 ± 0.004 *	2.040 ± 0.004
OPL	1.480 ± 0.002	1.475 ± 0.002	1.480 ± 0.002
ONL+IS	1.757 ± 0.008	1.765 ± 0.007	1.770 ± 0.008
OS	1.711 ± 0.004	1.717 ± 0.004	1.716 ± 0.005
RPE	1.659 ± 0.002	1.657 ± 0.002	1.660 ± 0.002
**Parafoveal Region**			
RNFL	1.447 ± 0.011 *	1.462 ± 0.011	1.492 ± 0.012 *
GCL+IPL	1.571 ± 0.003	1.568 ± 0.002	1.565 ± 0.003
GCC	3.017 ± 0.011	3.029 ± 0.010	3.057 ± 0.011 *
INL	1.670 ± 0.002	1.673 ± 0.002	1.674 ± 0.002
OPL	1.514 ± 0.002 *	1.505 ± 0.002 ‡	1.507 ± 0.002
ONL+IS	1.785 ± 0.007	1.793 ± 0.006	1.799 ± 0.007
OS	1.699 ± 0.005	1.708 ± 0.005	1.707 ± 0.005
RPE	1.674 ± 0.002	1.672 ± 0.002	1.672 ± 0.002
**Perifoveal Region**			
RNFL	1.461 ± 0.005	1.459 ± 0.005	1.468 ± 0.006
GCL+IPL	1.612 ± 0.002 ‡	1.605 ± 0.002	1.598 ± 0.003 *
INL	3.072 ± 0.005	3.063 ± 0.005	3.067 ± 0.005
OPL	1.681 ± 0.002 *	1.684 ± 0.002 *	1.683 ± 0.002
ONL+IS	1.522 ± 0.002 *	1.516 ± 0.002	1.515 ± 0.002
OS	1.796 ± 0.006 *	1.808 ± 0.005 *	1.814 ± 0.006
RPE	1.692 ± 0.004	1.706 ± 0.003	1.708 ± 0.004
GCC	1.676 ± 0.002	1.673 ± 0.002	1.674 ± 0.002

Abbreviations: SD (standard deviation); H (Healthy subjects group); MSON- (eyes of patients with multiple sclerosis without optic neuritis in medical history); MSON+ (eyes of patients with multiple sclerosis with optic neuritis in medical history); GCC (ganglion cell complex. the RNFL and GCL+IPL layers together)

* 0.001<p<0.05 and

‡ p<0.001 (Mixed-model analysis ANOVA) between H and MSON+ (see H column). H and MSON- (see MSON- column) and between MSON- and MSON+ (see MSON+ column)

### Layer index

The layer index values were significantly lower in the MSON- and MSON+ groups compared to the healthy controls, and also in the MSON+ versus MSON- comparisons in the RNFL, GCL+IPL and GCC in the whole macular, parafoveal and perifoveal regions. In the foveal region the GCL+IPL was significantly thinner in MSON+ compared to the other two groups, and the same was observed for the GCC in the MSON+ and H groups. (Table 5.)

**Table 5:** Distribution statistics of the layer index (a.u) of intraretinal layers by study group, represented as means ±SD.

Layer index	Healthy	MSON-	MSON+
	H vs. MSON+	H vs. MSON-	MSON+ vs MSON-
**Across All Macular Regions**			
RNFL	11.06 ± 0.25 ‡	9.28 ± 0.25 ‡	7.90 ± 0.27 ‡
GCL+IPL	13.49 ± 0.33 ‡	11.32 ± 0.32 ‡	9.84 ± 0.34 ‡
GCC	24.56 ± 0.55 ‡	20.62 ± 0.53 ‡	17.70 ± 0.57 ‡
INL	4.37 ± 0.13 *	3.97 ± 0.13 *	3.98 ± 0.14
OPL	6.39 ± 0.20 *	5.84 ± 0.20	5.57 ± 0.21
ONL+IS	7.49 ± 0.23 *	6.67 ± 0.22 *	6.83 ± 0.24
OS	6.05 ± 0.14	5.70 ± 0.13	5.67 ± 0.14
RPE	6.47 ± 0.08	6.55 ± 0.07	6.45 ± 0.08
**Foveolar Region**			
ONL+IS	9.03 ± 0.32 *	7.88 ± 0.31 *	7.87 ± 0.34
OS	6.55 ± 0.13 *	6.04 ± 0.12 *	6.16 ± 0.13
RPE	7.85 ± 0.11 *	7.9 ± 0.11 *	7.67 ± 0.11
**Foveal Region**			
RNFL	5.28 ± 0.18 *	4.82 ± 0.17	4.49 ± 0.19
GCL+IPL	13.98 ± 0.40 ‡	12.25 ± 0.38 *	10.63 ± 0.41 ‡
GCC	19.25 ± 0.55 ‡	17.06 ± 0.53 *	15.13 ± 0.57 *
INL	4.45 ± 0.15	4.09 ± 0.15	4.04 ± 0.16
OPL	9.19 ± 0.35 *	8.41 ± 0.34	7.59 ± 0.37
ONL+IS	8.51 ± 0.27	7.55 ± 0.26 *	7.73 ± 0.28
OS	6.38 ± 0.18	6.07 ± 0.17	6.05 ± 0.19
RPE	6.73 ± 0.09	6.81 ± 0.09	6.64 ± 0.10
**Parafoveal Region**			
RNFL	9.22 ± 0.21 ‡	7.92 ± 0.21 ‡	6.86 ± 0.22 ‡
GCL+IPL	15.94 ± 0.41 ‡	13.36 ± 0.39 ‡	11.60 ± 0.43 ‡
GCC	25.17 ± 0.58 ‡	21.29 ± 0.56 ‡	18.43 ± 0.61 ‡
INL	4.83 ± 0.15	4.43 ± 0.15	4.46 ± 0.16
OPL	6.12 ± 0.22	5.61 ± 0.21	5.55 ± 0.23
ONL+IS	7.65 ± 0.24	6.84 ± 0.24 *	7.05 ± 0.26
OS	5.88 ± 0.19	5.63 ± 0.18	5.64 ± 0.20
RPE	6.24 ± 0.09	6.28 ± 0.08	6.17 ± 0.09
**Perifoveal Region**			
RNFL	13.39 ± 0.32 ‡	10.96 ± 0.32 ‡	9.14 ± 0.34 ‡
GCL+IPL	12.41 ± 0.32 ‡	10.27 ± 0.31 ‡	8.93 ± 0.33 ‡
GCC	25.81 ± 0.60 ‡	21.24 ± 0.57 ‡	18.02 ± 0.62 ‡
INL	4.17 ± 0.12 *	3.79 ± 0.12 *	3.81 ± 0.13
OPL	4.86 ± 0.15 *	4.48 ± 0.14	4.45 ± 0.15
ONL+IS	6.64 ± 0.21	5.94 ± 0.21 *	6.11 ± 0.23
OS	5.83 ± 0.14	5.47 ± 0.13	5.46 ± 0.14
RPE	6.21 ± 0.08	6.33 ± 0.08	6.26 ± 0.08

Abbreviations: SD (standard deviation); H (Healthy subjects group); MSON- (eyes of patients with multiple sclerosis without optic neuritis in medical history); MSON+ (eyes of patients with multiple sclerosis with optic neuritis in medical history); GCC (ganglion cell complex. the RNFL and GCL+IPL layers together)

* 0.001<p<0.05 and

‡ p<0.001 (Mixed-model analysis ANOVA) between H and MSON+ (see H column). H and MSON- (see MSON- column) and between MSON- and MSON+ (see MSON+ column)

### Total reflectance

The total reflectance was found significantly different in the RNFL and GCC in both disease groups in the whole macular, the parafoveal and perifoveal regions, and the same was true for the GCL+IPL between the three groups in the parafoveal and perifoveal regions. In the foveal region a significant difference was observed in GCL+ IPL in MSON+ compared to the other groups, and also in the GCC complex for the comparison between the MSON+ and H groups. (Table 6.)

**Table 6:** Distribution statistics of the total reflectance (dB) with compensation to RPE layer reflectance of intraretinal layers by study group, represented as means ±SD.

Total reflectance	Healthy	MSON-	MSON+
	H vs. MSON+	H vs. MSON-	MSON+ vs MSON-
**Across All Macular Regions**			
RNFL	21.52 ± 0.26 ‡	19.90 ± 0.26 ‡	18.54 ± 0.28 ‡
GCL+IPL	22.50 ± 0.29 ‡	20.76 ± 0.28 ‡	19.62 ± 0.30 ‡
GCC	45.72 ± 0.53 ‡	42.14 ± 0.51 ‡	39.4 ± 0.56 ‡
INL	13.03 ± 0.30	12.10 ± 0.29 *	12.17 ± 0.31
OPL	14.30 ± 0.29 *	13.52 ± 0.28 *	13.48 ± 0.30
ONL+IS	16.98 ± 0.32	15.99 ± 0.31 *	16.28 ± 0.34
OS	15.89 ± 0.21 *	15.3 ± 0.20 *	15.20 ± 0.22
RPE	16.43 ± 0.10	16.58 ± 0.09	16.45 ± 0.10
**Foveolar Region**			
ONL+IS	19.18 ± 0.34 *	18.06 ± 0.33 *	18.05 ± 0.36
OS	16.55 ± 0.17 *	15.86 ± 0.17 *	16.00 ± 0.18
RPE	18.11 ± 0.12	18.19 ± 0.12	17.93 ± 0.13
**Foveal Region**			
RNFL	14.87 ± 0.32 *	14.01 ± 0.31	13.41 ± 0.33
GCL+IPL	23.30 ± 0.27 ‡	22.11 ± 0.27 *	20.92 ± 0.29 ‡
GCC	38.16 ± 0.56 ‡	36.12 ± 0.54 *	34.33 ± 0.59 *
INL	13.29 ± 0.30	12.57 ± 0.29	12.50 ± 0.32
OPL	19.42 ± 0.36 *	18.61 ± 0.35	17.82 ± 0.38
ONL+IS	18.84 ± 0.29	17.85 ± 0.28 *	18.08 ± 0.31
OS	16.41 ± 0.25	15.99 ± 0.25	15.94 ± 0.27
RPE	16.88 ± 0.11	17.01 ± 0.11	16.82 ± 0.12
**Parafoveal Region**			
RNFL	19.88 ± 0.26 ‡	18.55 ± 0.25 ‡	17.34 ± 0.27 ‡
GCL+IPL	24.58 ± 0.26 ‡	23.05 ± 0.25 ‡	21.80 ± 0.27 ‡
GCC	44.47 ± 0.49 ‡	41.61 ± 0.47 ‡	39.11 ± 0.51 ‡
INL	14.14 ± 0.28	13.44 ± 0.27	13.47 ± 0.29
OPL	16.17 ± 0.30	15.41 ± 0.29	15.35 ± 0.32
ONL+IS	18.12 ± 0.29	17.23 ± 0.29 *	17.48 ± 0.31
OS	15.86 ± 0.28	15.52 ± 0.27	15.47 ± 0.30
RPE	16.41 ± 0.11	16.5 ± 0.11	16.37 ± 0.12
**Perifoveal Region**			
RNFL	23.20 ± 0.29 ‡	21.34 ± 0.28 ‡	19.83 ± 0.30 ‡
GCL+IPL	22.50 ± 0.29 ‡	20.76 ± 0.28 ‡	19.62 ± 0.30 ‡
GCC	45.72 ± 0.53 ‡	42.14 ± 0.51 ‡	39.40 ± 0.56 ‡
INL	13.03 ± 0.30	12.10 ± 0.29 *	12.17 ± 0.31
OPL	14.30 ± 0.29 *	13.52 ± 0.28 *	13.48 ± 0.30
ONL+IS	16.98 ± 0.32	15.99 ± 0.31 *	16.28 ± 0.34
OS	15.89 ± 0.21 *	15.30 ± 0.20 *	15.20 ± 0.22
RPE	16.43 ± 0.10	16.58 ± 0.09	16.45 ± 0.10

Abbreviations: SD (standard deviation); H (Healthy subjects group); MSON- (eyes of patients with multiple sclerosis without optic neuritis in medical history); MSON+ (eyes of patients with multiple sclerosis with optic neuritis in medical history); GCC (ganglion cell complex. the RNFL and GCL+IPL layers together)

* 0.001<p<0.05 and

‡ p<0.001 (Mixed-model analysis ANOVA) between H and MSON+ (see H column). H and MSON- (see MSON- column) and between MSON- and MSON+ (see MSON+ column)

## Discussion

Optical coherence tomography has been used extensively to describe structural changes in the retina. In case of patients with multiple sclerosis, significant changes of retinal structure were previously described by histology and by Fourier-domain OCT (FD-OCT) image segmentation, not only in the inner, but also in the outer retinal layers. [36–39] Green et al. conducted postmortem evaluations in 82 patients with MS. They found histological changes not only in the ganglion cell layer but also in the inner nuclear layer, comprising the bipolar and amacrine cells. The atrophy of these layers was pressumed to be related to trans-synaptic degeneration and not the observed focal vascular inflammatory changes. [36] Interestingly, microcystic macular edema has been recently described in the INL in a small, severely impaired subset of MS patients with an unclear etiology. [10, 40, 41] Saidha et al. described the thinning of the outer retina additionally to the inner retinal changes using OCT image segmentation that appeared independently of inner retinal and optic nerve pathology, suggesting a primary retinal process in the background. [37–39] This was also supported by a small study employing adaptive optics scanning laser ophthalmoscopy imaging where–although only in one subject with MS—the thinning of the outer retina was suggested to be due to the cone density decrease caused by optic neuropathy. [42]

The inner retinal changes appear to be in connection with central nervous system changes, e.g. intracranial or brain substructure volume reduction (i.e. brain atrophy). [43–45] Therefore, OCT may provide a possibility to better understand the neurobiological changes in neurodegenerative diseases such as MS and may help to develop both diagnostic and prognostic biomarkers that can predict clinical progress.

Optical changes of the retina have been described earlier in glaucoma, a neurodegenerative disease characterized by the loss of inner retinal structure that is very similar to that observed in MS. It has been previously shown that the reflectance of the RNFL layer shows changes in eyes with glaucoma in human patients [46, 47] and in rat eyes with elevated IOP and glaucoma, as well. [21] According to the work of Huang et al. the thinning of the RNFL observed in a rat model of glaucoma is preceded by reflectance changes that could be explained by the changes in the optical properties of the extracellular and/or cytoplasmic constituents before cytostructural damage occurs. [21]

To our knowledge, this is the first report describing optical differences observed in the retinal tissue of patients with MS. Additionally, we compared various optical parameters to thickness in order to reveal which parameter could be the best predictor of existing pathology.

The parameters that revealed significant differences between study groups were the thickness, layer index and total reflectance, mostly in eyes with MSON in the previous history, indicating optical changes in the inner retina following optic neuritis. The findings of the significant thinning of the RNFL, GCL+IPL and GCC complex in MS patients compared to healthy and MSON+ subjects compared to MSON- patients, are in agreement with our earlier results and the results of previous studies. [8, 37–39, 41, 48] More interestingly, there were significant changes in the layer index and total reflectance also in the eyes without ON in the previous history. At the same time, the foveal region showed differences only with missed significance (very small p values close to 0.001) in the RNFL, GCL+IPL and GCC layers in all above parameters (except the fractal dimension), which may be explained by the compensation mechanism of the OCTRIMA software that partly affects this region and uniforms the segmentation of this area.

These results draw attention to the structural and optical changes in the macular area in MS even without ON supporting the previous view of ongoing neurodegeneration also present in the retina.[8, 43, 49, 50]

For the contrast, a significant reduction was observed only when looking at the entire macular region, between MSON+ versus healthy and MSON- eyes in the deeper inner retinal layers (INL and OPL), although these differences were not expressed in other regions and parameters. These previous findings suggest that atrophy stops at the level of the INL, which has been referred as a barrier to trans-synaptic axonal degeneration.[36, 51, 52] Also, they are in agreement with the view that MS is both a demyelinating and neurodegenerative disease. [48] We observed significant difference in the contrast and fractal dimension parameters in the INL and OPL in the whole macular, foveal and parafoveal regions, which in partly agrees with previous reports. [37, 39, 53]

The outer retina showed no significant differences between the groups which was in contrast with our earlier report showing significant fractal dimension changes in diabetic patients where, similarly, neurodegeneration was a proposed mechanism in the background of the observed changes. [54] This could point to the fact of a different disease mechanism at the level of the photoreceptors.

The observed differences in optical properties in our study can be related both to circulational or inflammatory alterations of the inner retina. There is only little evidence about the changes in microcirculation of the retina in MS. A recent study by Wang et al. using OCT angiography showed significantly reduced flow index around the optic nerve head in eyes after optic neuritis compared to healthy controls but no differences were shown in the parafoveal circulation. [55] Although this may suggest that our observations were most possibly not influenced by alterations in macular microcirculation, recent results also using hemodynamic information have revealed retinal impaired microcirculation in the macular region. [53, 56–58] Therefore, further investigation is needed to better characterize the structure-function relationship in MS. On the other hand, inflammation may presumably be present also in the retina, possibly supported by the “inside-out” theory of MS, namely the migration of autoreactive T cells across the blood-brain barrier from the systemic circulation leading to inflammation. [55] The blood-retina barrier is very similar to the blood-brain barrier and indeed, the histological study by Green et al. described inflammatory cellular infiltrates surrounding retinal veins in the connective tissue of the retinal nerve fiber layer and GCL in 29% of the relapsing remitting and secondary progressive multiple sclerosis eyes. [36] It should be noted, however, that the observed inflammation was more localized (i.e not in all vessels or the entire retina) than the neurodegeneration observed which makes the direct correlation with our observed trends questionable in terms of revealing inflammatory alterations present or not in our study data. However, taking into account that the inner capillary network lies in the GCL and the outer capillary network runs from the IPL to the OPL through the INL, the significant differences observed between the study groups when analyzing reflectance and texture descriptors of the RNFL and GCL+IPL complex should be further explored in a larger study and correlated to microvasculature measurements (e.g. blood flow velocity and perfusion) using advanced optical imaging technologies. [55, 59, 60] Investigating these two capillary networks in relation to structural, optical and functional measures may provide a much better insight to determine the role of the retinal microcirculation (i.e capillaries, arterioles and venules) in the increased risk of progression of multiple sclerosis in the presence of vascular comorbidities. [61, 62]

Our study has certain limitations, one of them being the small number of subjects included. This study was aimed to see the possible usefulness of texture and optical property measurements in MS, but further work is warranted on a larger group of patients in the future. Second, the OCT device used in this study was time-domain OCT, which has a lower image resolution compared to latest SD-OCT technology, but it was a standard when the study was initiated in 2010. In addition, we have previously shown a high level of repeatability for both the RNFL and the GCL+IPL layers in our studies using OCTRIMA on TD-OCT images where the axial range (depth range) is 2 mm, consequently the 1024 axial pixels have a spacing of 2 μm (i.e. axial pixel resolution is 2 mm/1024) in depth. [23] Though our analysis is not affected by interpolation errors because the analysis was performed per ROI instead of using the standard ETDRS maps, higher scanning speeds could permit a higher sampling density of the scanned region, requiring a more accurate representation of the retinal structure. Third, study data were not stratified in terms of age and disease duration for the ON+ group when compared to healthy subjects. We believe this does not affect our results largely as disease duration and age were comparable in the three groups.

Although optical properties of the retinal tissue are not standardized measures for detecting pathological changes of the retina, in contrast to thickness measurements, reflectance-based measures are direct measures obtained from OCT images. Therefore, we expect these additional properties along with thickness information could facilitate a better diagnosis of retinal diseases. It is also worth to note that only qualified OCT images that had signal levels above the manufacturer recommended cutoff (i.e. SS higher than 6) and also had an SQF of 1 were used in the analysis. In addition, signal normalization was implemented to reduce the difference in measurements and minimize systematic technical variance and bias before assessing biological variability in study eyes. Therefore, normalization allowed us to compare the features of all of the images, ruling out differences in signal strength and mean background values. Also, we have shown previously that our custom-built segmentation algorithm provides comparable results to those obtained by SD-OCT and therefore we expect the trends reported here to be replicated by advanced OCT devices. [41] Further studies are warranted.

## Conclusion

In summary, it seems that texture and optical properties of the retina derived from OCT images may provide a useful additional tool in the hands of clinicians for the assessment of neurodegeneration and neuronal loss-related changes occurring in MS. This may help the better differentiation of eyes with ON and more precise targeting of potential therapeutic interventions and could also be useful in the follow-up of MS patients. However, in order to prove this, a larger set of patients needs to be involved taking advantage of state-of-the art imaging technology and segmentation procedures.

## Supporting Information

S1 Table Demographical data and results of visual acuity test of the patients with multiple sclerosis (MS, Table A) and control subjects (Table B.). The disease duration and time from the last MS associated optic neuritis (MSON) was measured by month. It was calculated from July if only the year of ON episode was known. In those cases, when the ON episode was at the year of examination, the time was than calculated from January.
(XLSX)

S2 Table Results of thickness measurements of intraretinal layer of the subjects. Sheet 1. Distribution statistics of the thickness (μm) of intraretinal layers by study group represented as means ± SD and p values of the comparison between study groups. Sheet 2–6. The results organized by study groups and regions.
(XLSX)

S3 Table Results of contrast calculations of intraretinal layer of the subjects. Sheet 1. Distribution statistics of the contrast (a.u.) of intraretinal layers by study group represented as means ± SD and p values of the comparison between study groups. Sheet 2–6. The results organized by study groups and regions.
(XLSX)

S4 Table Results of fractal dimension calculations of intraretinal layer of the subjects. Sheet 1. Distribution statistics of the fractal dimension (a.u.) of intraretinal layers by study group represented as means ± SD and p values of the comparison between study groups. Sheet 2–6. The results organized by study groups and regions.
(XLSX)

S5 Table Results of layer index calculations of intraretinal layer of the subjects. Sheet 1. Distribution statistics of the layer index (a.u.) of intraretinal layers by study group represented as means ± SD and p values of the comparison between study groups. Sheet 2–6. The results organized by study groups and regions.
(XLSX)

S6 Table Results of total reflectance measurements of intraretinal layer of the subjects. Sheet 1. Distribution statistics of the total reflectance (dB) of intraretinal layers by study group represented as means ± SD and p values of the comparison between study groups. Sheet 2–6. The results organized by study groups and regions. Abbreviations used in the tables: std (standard deviation); H (Healthy subjects group); MSON- (eyes of patients with multiple sclerosis without optic neuritis in medical history); MSON+ (eyes of patients with multiple sclerosis with optic neuritis in medical history); RNFL (retinal nerve fiber layer); GCL+IPL, ganglion cell layer and inner plexiform layer complex; GCC (ganglion cell complex. the RNFL and GCL+IPL layers together); INL, inner nuclear layer; OPL, outer plexiform layer; ONL+IS, combined outer nuclear layer and inner segment of photoreceptors; OS, outer segment of photoreceptors; RPE, retinal pigment epithelial layer.
(XLSX)
